# Supplementary material for: Crym-positive striatal astrocytes gate perseverative behaviour
Source: Nature. 2024 Feb 28;627(8003):358–66. doi: 10.1038/s41586-024-07138-0 (PMC10937394; doi:10.1038/s41586-024-07138-0)
Supplement: Supplementary file 1 — Raw western blots for Crym-BioID2 validation. [file 41586_2024_7138_MOESM1_ESM.pdf]

nature portfolio

https://doi.org/10.1038/s41586-024-07138-0

Supplementary information

Crym-positive striatal astrocytes gate perseverative behaviour

In the format provided by the authors and unedited

Corresponding to Extended data Figure 13f

Streptavidin-HRP

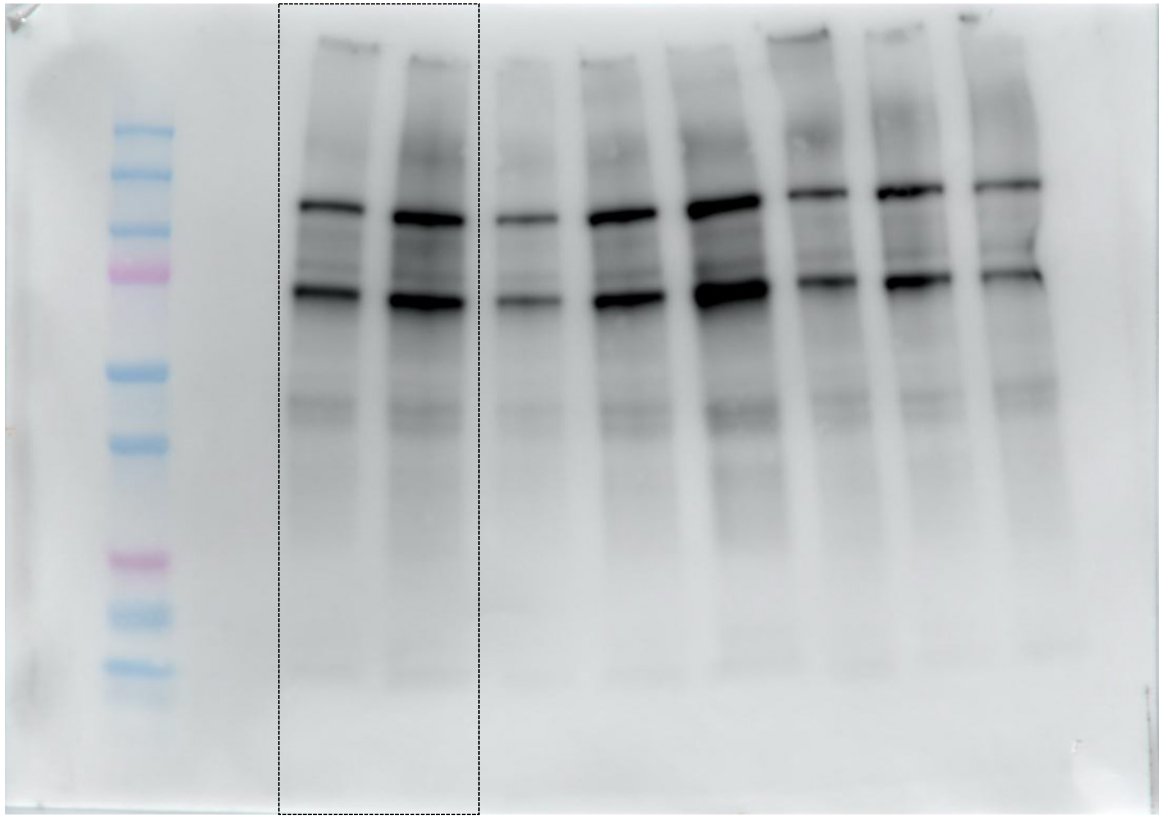

Beta-actin

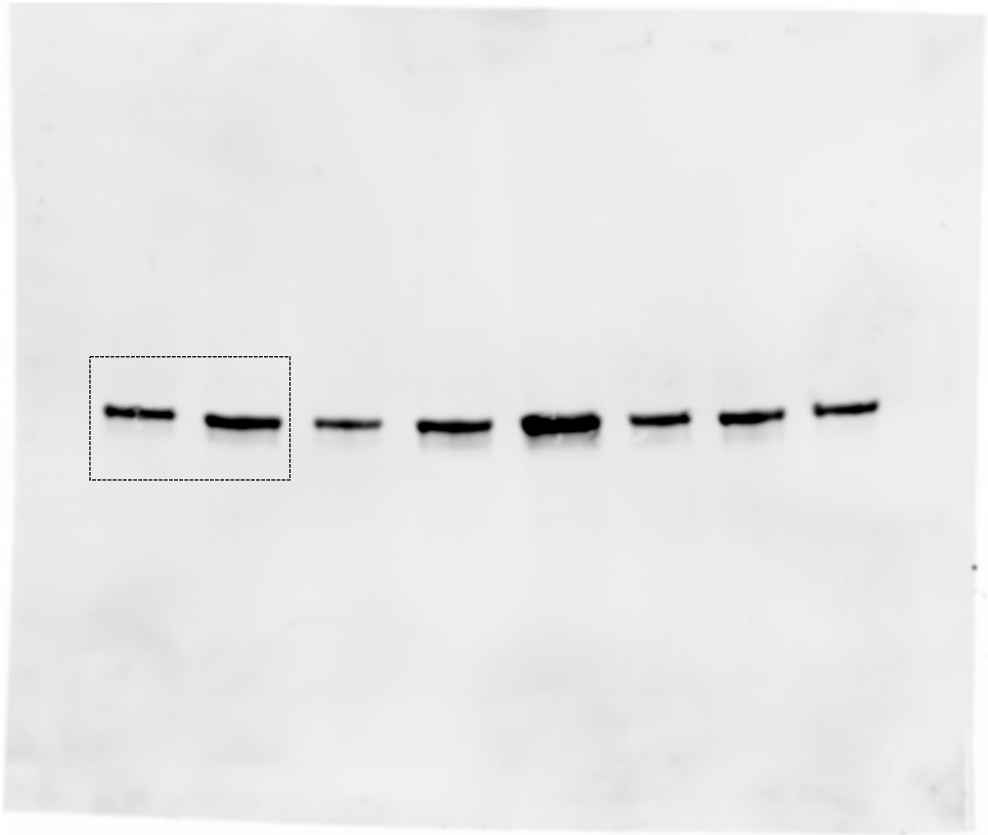

Supplementary Fig 1: Both images represent the same blot. Left image shows chemiluminescence signal, while right blot show fluorescence. Please see methods for further details.
