# Supplementary material for: Crym-positive striatal astrocytes gate perseverative behaviour
Source: Nature. 2024 Feb 28;627(8003):358–66. doi: 10.1038/s41586-024-07138-0 (PMC10937394; doi:10.1038/s41586-024-07138-0)
Supplement: Supplementary file 2 — Reporting Summary [file 41586_2024_7138_MOESM2_ESM.pdf]

nature portfolio

Corresponding author(s): Baljit S. Khakh

Last updated by author(s): Dec 31, 2023

Reporting Summary

Nature Portfolio wishes to improve the reproducibility of the work that we publish. This form provides structure for consistency and transparency in reporting. For further information on Nature Portfolio policies, see our Editorial Policies and the Editorial Policy Checklist.

Statistics

For all statistical analyses, confirm that the following items are present in the figure legend, table legend, main text, or Methods section.

n/a	Confirmed	
☐	☒	The exact sample size (*n*) for each experimental group/condition, given as a discrete number and unit of measurement
☐	☒	A statement on whether measurements were taken from distinct samples or whether the same sample was measured repeatedly
☐	☒	The statistical test(s) used AND whether they are one- or two-sided<br>*Only common tests should be described solely by name; describe more complex techniques in the Methods section.*
☐	☒	A description of all covariates tested
☐	☒	A description of any assumptions or corrections, such as tests of normality and adjustment for multiple comparisons
☐	☒	A full description of the statistical parameters including central tendency (e.g. means) or other basic estimates (e.g. regression coefficient) AND variation (e.g. standard deviation) or associated estimates of uncertainty (e.g. confidence intervals)
☐	☒	For null hypothesis testing, the test statistic (e.g. *F*, *t*, *r*) with confidence intervals, effect sizes, degrees of freedom and *P* value noted<br>*Give P values as exact values whenever suitable.*
☒	☐	For Bayesian analysis, information on the choice of priors and Markov chain Monte Carlo settings
☒	☐	For hierarchical and complex designs, identification of the appropriate level for tests and full reporting of outcomes
☐	☒	Estimates of effect sizes (e.g. Cohen's *d*, Pearson's *r*), indicating how they were calculated

Our web collection on statistics for biologists contains articles on many of the points above.

Software and code

Policy information about availability of computer code

Data collection	Proteomics: The spectra were collected using data dependent acquisition on Orbitrap Fusion Lumos Tribrid mass spectrometer (Thermo Fisher Scientific) with an MS1 resolution of 120,000 followed by sequential MS2 scans at a resolution of 15,000. Data generated by LC-MS/MS were searched using the Andromeda search engine integrated into the MaxQuant (Cox et al,. 2008) bioinformatic pipelines against the Uniprot Mus musculus reference proteome (UP000000589 9606) and then filtered using a "decoy" database-estimated false discovery rate (FDR) < 1%. Label-free quantification (LFQ) was carried out by integrating the total extracted ion chromatogram (XIC) of peptide precursor ions from the MS1 scan. These LFQ intensity values were used for protein quantification across samples. Label-free quantification was carried out by the MaxQuant software with integrated search engine, Andromeda (https://www.maxquant.org/).<br>Metabolomics: Derivatized metabolites were analyzed using a DB-35MS column (30m × 0.25mm i.d. × 0.25µm, Agilent J&W Scientific) in an Agilent 7890A gas chromatograph coupled to a 5975C mass spectrometer.<br>RNA-seq: Sequencing was performed on Illumina HiSeq 4000. Reads were aligned to the mouse mm10 reference genome using the STAR spliced read aligner v2.7.5c (Dobin et al, 2013)<br>sc-RNA Seq: sc-RNA seq data was processed with the 10X genomics platform. Single cell libraries were generated and sequenced on the Illumina NextSeq500 sequencer.<br>Behavior: Open field, elevated plus maze, and lickometer testing was collected and analyzed simultanously by Anymaze (v6.3 Stoelting Co. Wooddale, IL, USA).<br>Calcium imaging: GECIquant sofware (v1.0) was used to analyze calcium signals in microdomains.<br>Imaging for IHC and RNA-scope was conducted on an Olympus FV3000 confocal microscope using Fluoview software (FV31S-SW, v2.61.243).<br>Electrophysiological recordings were performed using pCLAMP11.2 (Axon Instruments) using a Multiclamp 700B amplifier (Axon instruments).<br>Analysis for electrophysiological recordings was conducted using ClampFit 10.7 software.<br>Western blot data was collected on a GE Amersham 680 imager.
Data analysis	Proteomics: Label-free quantification was carried out by the MaxQuant software with integrated search engine, Andromeda (https://www.maxquant.org/). Differential protein expression and enrichment analysis was conducted with Bioconductor R package, limma v 3.54 (https://bioconductor.org/packages/release/bioc/html/limma.html). Protein network visualization, including STRING analysis was conducted

with Cytoscape v 3.8 (https://apps.cytoscape.org/apps/stringapp). The artMS package v 1.16 (https://bioconductor.riken.jp/packages/3.8/bioc/html/artMS.html) was used to re-format the maxquant results (evidence.txt file), to make them compatible with SAINTexpress program.
SAINT protein interaction probability scoring was done through (http://saint-apms.sourceforge.net/Main.html).
RNA-seq: Differential gene expression and enrichment analysis used R package limmaVoom v 3.36 to process RNA counts (https://rdrr.io/bioc/limma/man/voom.html).
IHC, RNA-scope, proximity, western blots: Microscopy data and western blot data was imported and analyzed on FIJI (ImageJ v 2.1) using the BioFormats importer for Olympus FV3000 acquired images.
sc-RNA Seq: Sequence reads were processed and aligned to the mouse genome using CellRanger 3.0 (10X Genomics). Processing and visualization were conducted with R-package Seurat (https://CRAN.R-project.org/package=Seurat, Satija Lab)
All data, unless otherwise stated were plotted with OriginPro 2017 (v 9.4.2)
Statistical analysis was conducted with OriginPro 2017 (v 9.4.2)

For manuscripts utilizing custom algorithms or software that are central to the research but not yet described in published literature, software must be made available to editors and reviewers. We strongly encourage code deposition in a community repository (e.g. GitHub). See the Nature Portfolio guidelines for submitting code & software for further information.

Data

Policy information about availability of data

All manuscripts must include a data availability statement. This statement should provide the following information, where applicable:

- Accession codes, unique identifiers, or web links for publicly available datasets
- A description of any restrictions on data availability
- For clinical datasets or third party data, please ensure that the statement adheres to our policy

The proteomic data are available at PRIDE with accession ID PXD040991. The UniProt reference proteome used was UniProt UP000000589 for Mus musculus. The RNA-seq data are available at GEO with accession ID GSE228506. scRNAseq data are available at GEO with accession ID GSE225741. Proteomic data are provided as Extended data Excel file 3. The analyzed RNA-seq data are provided as Extended data Excel files 1 and 2. Statistical tests for all figures are provided in Extended data Excel file 4.

Field-specific reporting

Please select the one below that is the best fit for your research. If you are not sure, read the appropriate sections before making your selection.

☒ Life sciences ☐ Behavioural & social sciences ☐ Ecological, evolutionary & environmental sciences

For a reference copy of the document with all sections, see nature.com/documents/nr-reporting-summary-flat.pdf

Life sciences study design

All studies must disclose on these points even when the disclosure is negative.

Sample size	Power analysis was conducted using values for power of 0.8 or higher and alpha of 0.1 or lower and an estimated effect size based on pilot data. Furthermore, group sizes were selected based on data from the use of similar models by our laboratory.
Data exclusions	No data was excluded from this manuscript
Replication	To verify the reproducibility of the experimental findings, all data collection was done in multiple batches comprising at least four replicates. The proteomic data analyses was conducted from 4 independently processed batches that each contained 8 mice for each experimental group (in all cases). Behavioral data was conducted as the mice became available from the breeding colony and each experiment/recording was done in 2-3 batches containing between 3-6 mice per group. All experiments were successfully replicated.
Randomization	For all experiments, the mice were randomly allocated to a group as they became available and of age from the breeding colony in alternation.
Blinding	For all analyses, the investigators were blinded to group allocation during data collection, as numerical mouse IDs were the only identifier used.

Reporting for specific materials, systems and methods

We require information from authors about some types of materials, experimental systems and methods used in many studies. Here, indicate whether each material, system or method listed is relevant to your study. If you are not sure if a list item applies to your research, read the appropriate section before selecting a response.

Materials & experimental systems

n/a	Involved in the study
☐	☒ Antibodies
☒	☐ Eukaryotic cell lines
☒	☐ Palaeontology and archaeology
☐	☒ Animals and other organisms
☒	☐ Human research participants
☒	☐ Clinical data
☒	☐ Dual use research of concern

Methods

n/a	Involved in the study
☒	☐ ChIP-seq
☒	☐ Flow cytometry
☒	☐ MRI-based neuroimaging

Antibodies

Antibodies used

Primaries:
mouse anti-μ-crystallin (Santa Cruz, #sc-376687, clone F-11, lot #E1018)
chicken anti-GFP (Abcam #ab13970)
mouse anti-NeuN (Millipore #MAB377, clone A60, lot #2884594)
guinea pig anti-NeuN (Synaptic system, #266004, lot#G-53)
rabbit anti-DARPP32 (Abcam, #ab40801, clone EP720Y, lot #1007414-1)
rabbit anti-S100ß (Abcam #ab41548)
rabbit anti-cFos (Synaptic system #226008, lot #108B5)
rabbit anti-RFP (Rockland #600–401-379, lot #48710)
rabbit anti-mCherry (Abcam, #ab167453)
rabbit anti-Opioid Receptor μ, pain (MOR1) (Millipore-sigma, #AB5511, lot #1007414-1)
chicken anti-Calbindin D-28K (Novus biologicals, #NBP2-50028)
chicken anti-GFAP (Abcam, #ab4674, lot #1012209-2)
rabbit anti-HA tag (Abcam, #ab9110, #)
mouse anti-HA tag (Biolegend, #901514)
rabbit anti-β-actin (Abcam, #ab8227)
rabbit anti-USP9X (Proteintech, #55054-1-AP, lot #00043048)
rabbit anti-MAP2 (Thermofisher scientific, #PA1-10005, lot #YG3995721)
guinea pig anti-RFP (Synaptic Systems, #390004)
rabbit anti-Sox9 (EMD Millipore, #AB5535, lot #3836442)
rabbit anti-Olig2 (EMD Millipore #AB9610, lot #3814881))
rabbit anti-Kir4.1 (Alomone labs, #APC-035, lot #AN1002)
rabbit anti-ATP1a2 (Proteintech, #16836-1-AP)
rabbit anti-GLT1 (Synaptic Systems, #250203, lot #1-10)
mouse anti-GABA (abcam, #ab86163, lot #GR3423846-5)
rabbit anti-MAOB (Thermofisher #PA5-28338, lot #YH4009980A)
rabbit anti-GAT3 (gift from the Brecha lab, UCLA)
rabbit anti-Capzb (Thermo Fisher, #PA5-83196)

Secondaries:
Goat anti-rabbit plus 647 (Invitrogen, A32733, lot #VC299350)
Alexa Fluor 405 goat anti-mouse (A31553, lot #2491371)
Alexa Fluor 488 goat anti-chicken (A11039, lot #2566343)
Alexa Fluor 488 goat anti-rabbit(A11008, lot #2420730))
Alexa Fluor 488 goat anti-mouse (A11001, lot #2610355)
Alexa Fluor 546 goat anti-mouse (A11030, lot #2155294)
Alexa Fluor 546 goat anti-rabbit (A11010, lot #2570547)
Alexa Fluor 647 goat anti-rabbit (A21244, lot #2497486)
Streptavidin, Alexa Fluor 488 conjugate (S11223, lot #18585036)
Streptavidin-HRP (Sigma, RABHRP3)
Donkey anti-guinea pig Cy3 (Jackson ImmunoResearch, #706-165-148, lot #159084)
IR-dye 800CW anti-rabbit (Li-Cor, #925-32211, lot #C80118-01)

Validation

The antibodies used in this manuscript have been validated and reproduced by our lab across at least 7 manuscripts by checking cell specificity, background signal, and noting antigen specificity using western blot techniques (Srinivasan et al., 2016; Chai et al., 2017, Nagai et al., 2019; Yu et al., 2020; Diaz-Castro et al., 2019; Endo et al., 2022, Gangwani et al., 2023). All Khakh lab manuscripts.

Animals and other organisms

Policy information about studies involving animals; ARRIVE guidelines recommended for reporting animal research

Laboratory animals

All animal experiments were conducted in accordance with the National Institutes of Health Guide for the Care and Use of Laboratory Animals and were approved by the Chancellor's Animal Research Committee at the University of California, Los Angeles. All mice were housed with food and water available ad libitum in a 12-hour light/dark environment. All animals were healthy with no obvious

behavioral phenotype, were not involved in previous studies, and were sacrificed during the light cycle. Data for experiments were collected from adult mice aged 9-15 weeks old. To characterize u-crystallin expression during development and ageing, mice were used between P0 and 22 months old. C57Bl/6NTac mice were maintained as an in-house breeding colony or purchased from Taconic Biosciences. CAG-Cas9 transgenic mice (B6J.129(Cg)-Gt(ROSA)26Sortm1.1(CAG-cas9*,-EGFP)Fezh/J, JAX Stock # 026179) and SAPAP3 -/- mice (B6.129-Dlgap3tm1Gfng/J, JAX Stock # 008733) were purchased from the Jackson Laboratory and maintained as breeding colonies at UCLA. SAPAP3 -/- mice were used at 6 months of age. Tg(Crym-EGFP)GF82Gsat(strain 012003-UCD) reporter mice were obtained from MMRRC and maintained as a breeding colony at UCLA. Both males and females were used in alternating batches.

Wild animals	The study did not involve wild animals
Field-collected samples	This study did not use field-collected samples
Ethics oversight	All experiments were conducted in accordance with the National Institutes of Health (NIH) Guide for the Care and Use of Laboratory Animals and were approved and overseen by the Chancellor's Animal Research Committee (ARC) at the University of California, Los Angeles (UCLA)

Note that full information on the approval of the study protocol must also be provided in the manuscript.
