# Supplementary material for: Crym-positive striatal astrocytes gate perseverative behaviour
Source: Nature. 2024 Feb 28;627(8003):358–66. doi: 10.1038/s41586-024-07138-0 (PMC10937394; doi:10.1038/s41586-024-07138-0)
Supplement: Supplementary file 4 — AAV constructs and Addgene IDs. [file 41586_2024_7138_MOESM4_ESM.pdf]

**AAV construct**	**Addgene ID #**	**Titer**
*AAV2/5 *U6*-sgRNA-Crym(x3)-*GfaABC$_1$D*-mCherry	200067	3×10^{13} gc/ml
*AAV2/5 *U6*-sgRNA-GFP-*GfaABC$_1$D*-mCherry	200068	2.1×10^{13} gc/ml
*AAV2/5 *GfaABC$_1$D*-*Crym*-EGFP	200080	2.5×10^{13} gc/ml
*AAV2/5 *GfaABC$_1$D*-jGCaMP8m	213010	2.2×10^{13} gc/ml
AAV1 *hSyn*-Chronos-GFP	59170	1.4×10^{13} gc/ml
AAV1 *hSyn*-hM4D(Gi)-mCherry	50475	2.4×10^{13} gc/ml
AAV1 *hSyn*-mCherry	114472	2.4×10^{13} gc/ml
AAV2/5 *GfaABC$_1$D*-Rpl22-HA	111811	2.1×10^{13} gc/ml
AAV 2/5 *GfaABC$_1$D*-*Crym*-BioID2-HA	200070	3.1×10^{13} gc/ml
AAV1 *CaMKIIa-hChR2(H134R)*-mCherry	26975	2×10^{13} gc/ml
AAV2/5 *GfaABC$_1$D*-tdTomato	44332	2×10^{13} gc/ml

Supplemental Table 1. Plasmids used in this study (* new AAV plasmids generated in this study).
